# NOT ENJOYING THE MEAL WAS ASSOCIATED WITH DEPRESSIVE SYMPTOMS, DESPITE THE SIZE OF SOCIAL CONNECTIONS

**DOI:** 10.1093/geroni/igz038.2366

**Published:** 2019-11-08

**Authors:** Mai Takase, Hiroshi Murayama, Sayaka Hirukawa, Tomoki Tanaka, Sachiko Ono, Sugimoto Minami, Mari Kimata

**Affiliations:** 1 The University of Tokyo, Institute of Gerontology, Bunkyo-ku, Tokyo, Japan; 2 Institute of Gerontology, The University of Tokyo, Tokyo, Japan; 3 The University of Tokyo, Bunkyo-ku, Tokyo, Japan; 4 Institute of Gerontology, The University of Tokyo, Japan, Tokyo, Japan

## Abstract

Conventional studies report that the enjoyment of the meal is related to likelihood of contracting depressive mood. The Japanese assisted living facilities currently support seniors build social connections to maintain their health and well-being, but psychological feeling during mealtime is often left unquestioned. Because seniors engage in conversation with tablemates while dining, the feeling during mealtime should not be ignored. This study aimed to explore the relationship among social connection, enjoyment during mealtime, and depressive mood. A cross-sectional questionnaire study was performed for independent residents at assisted living facility in Kanagawa Prefecture, Japan. The size of social connection (the number of facility residents that one can easily talk to) and enjoyment during the meal were assessed by a single item, respectively. The 15-item Geriatric Depression Scale was used to measure depressive symptoms. The analysis included 190 questionnaires. A logistic regression analysis showed that enjoyment during mealtime was associated with less likelihood of depressive symptoms, but the size of social connection was not, after adjusting for socio-demographics and health conditions. Moreover, a significant interaction between social connection and enjoyment during mealtime was observed. This indicated that greater size of social connection was inversely related to depressive symptoms among those who enjoyed the meal; however, among those who did not enjoy the meal, the likelihood of depressive symptoms were stably higher despite the size of social connection. The findings suggested that along with helping seniors build social connection, care takers should focus on improving the dining environment of seniors.

